# Torsion of a giant epididymal cyst in a pediatric patient: a rare cause of acute scrotal pain—case report and literature review

**DOI:** 10.3389/fped.2025.1605254

**Published:** 2025-05-26

**Authors:** Chenghao Zhanghuang, Jinrong Li, Na Long, Qiong Duan, Fengming Ji, Yucheng Xie, Zhen Yang, Bing Yan

**Affiliations:** ^1^ Department of Urology, Kunming Children’s Hospital, Children’s Hospital Affiliated to Kunming Medical University, Kunming, China; ^2^ Yunnan Key Laboratory of Children’s Major Disease Research, Yunnan Province Clinical Research Center for Children’s Health and Disease, Kunming Children’s Hospital Children’s Hospital Affiliated to Kunming Medical University, Kunming, China; ^3^ Special Need Ward, Kunming Children’s Hospital, Children’s Hospital Affiliated to Kunming Medical University, Kunming, China; ^4^ Department of Pathology, Kunming Children’s Hospital, Children’s Hospital Affiliated to Kunming Medical University, Kunming, China; ^5^ Department of Oncology, Yunnan Children Solid Tumor Treatment Center, Kunming Children’s Hospital, Children’s Hospital Affiliated to Kunming Medical University, Kunming, China

**Keywords:** epididymal cyst torsion, acute scrotum, mesonephric duct remnant, surgical misdiagnosis, ultrasonography

## Abstract

Torsion of an enlarged epididymal cyst (EC) represents an uncommon yet critical differential diagnosis in acute scrotal emergencies. Large ECs, particularly those localized to the epididymal head, demonstrate diminished anatomical stability due to their superior positioning and increased mobility, rendering them susceptible to torsion under predisposing conditions. This mechanical disruption precipitates elevated intracystic pressure, compromised vascular perfusion, and subsequent infective complications, necessitating urgent surgical intervention. We present a case of a 12-year-old male who presented with left scrotal swelling and pain persisting for 48 h. Initial clinical evaluation suggested spermatic cord torsion concomitant with hydrocele, prompting emergency exploration. Intraoperative findings unexpectedly revealed a torsioned giant cyst (5.2 × 3.8 cm) originating from the left epididymal head. Successful detorsion followed by complete cyst excision was performed while preserving testicular integrity and epididymal architecture. Histopathological analysis confirmed the lesion as a mesonephric duct remnant cyst.

## Introduction

Epididymal cysts (ECs) are benign cystic lesions frequently observed in adults but relatively uncommon in the pediatric population (1). Although the precise pathogenesis of ECs remains unclear, some studies suggest that congenital anomalies caused by hormonal disturbances during embryonic development may contribute to their formation (2). Most EC cases are asymptomatic and are typically detected incidentally through ultrasonography, requiring no intervention unless complications such as torsion, infection, or trauma occur, which may manifest as acute testicular pain necessitating surgical or medical management (1, 3). Notably, EC torsion represents an exceptionally rare clinical entity, with only 12 cases documented in the literature to date, to the best of our knowledge.

## Case report

A 12-year-old boy was admitted for “left scrotal swelling and pain for 2 days.” He had intermittent left scrotal swelling and severe pain without obvious cause 2 days prior. He had no fever or vomiting. An ultrasound at a local hospital showed a cystic anechoic area above the left testis, suggesting hydrocele. The family declined surgery. His left scrotal redness, swelling, and pain worsened, leading to an emergency visit at our hospital. An ultrasound revealed a nodular image in the left spermatic cord, possibly due to torsion of a funicular hydrocele. He was admitted for “left scrotal swelling and pain pending investigation.”

**Figure 1:**
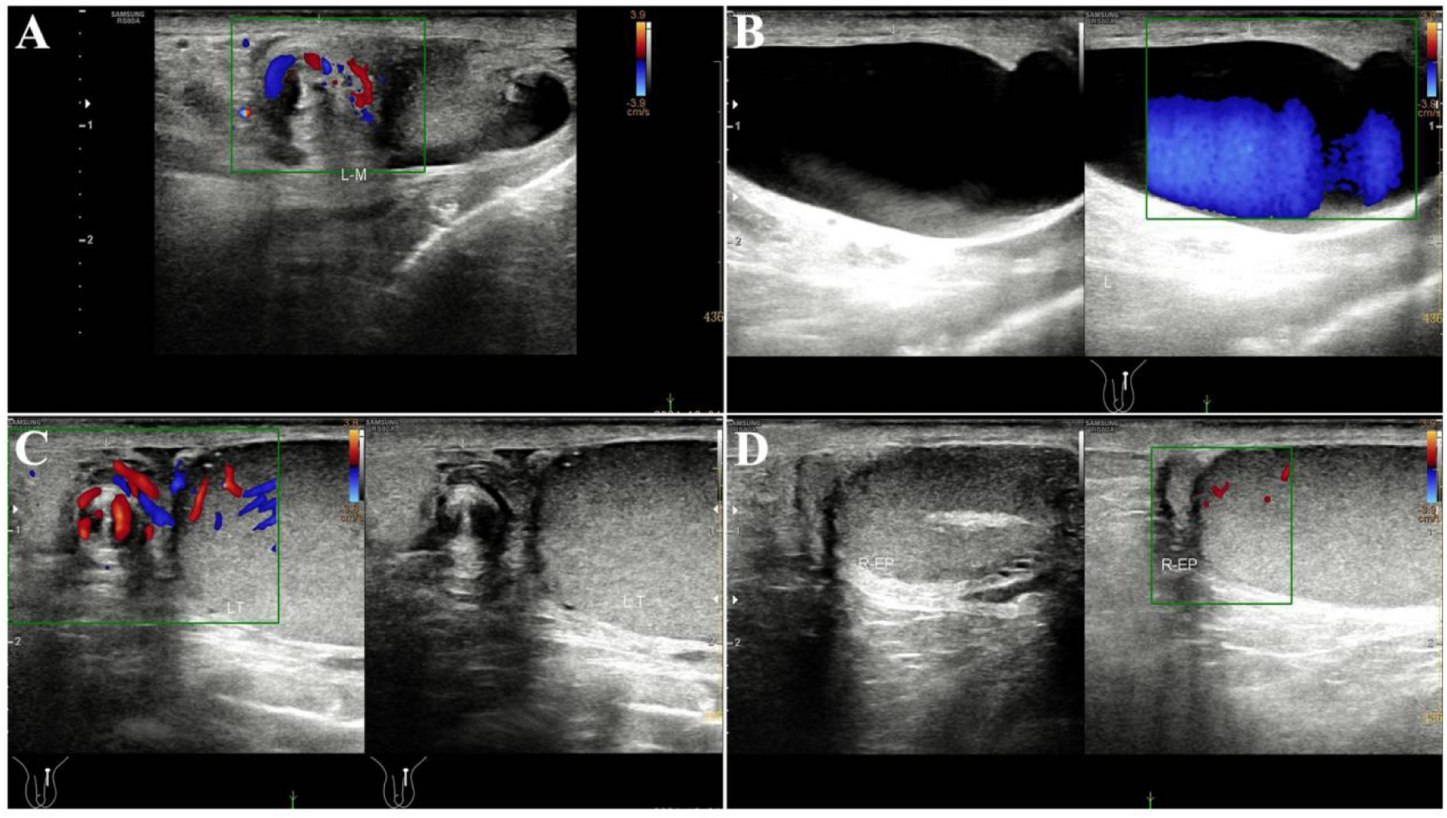
Preoperative emergency inguinal and scrotal testicular ultrasound findings; **(A)** spermatic cord two-dimensional image shows a spiral sign, and CDFI shows spiral blood flow signals; **(B)** spermatic cord hydrocele; **(C)** spermatic cord torsion, but blood flow signals are visible in the spermatic cord and testis; **(D)** blood flow signals are visible in the epididymal head and testis.

On specialized examination, his left scrotum was swollen and slightly red, with a cystic mass near the testis that was tender, smooth, soft, and poorly mobile. The right testis was normal in size and without tenderness. Both testes had normal cremasteric reflexes. A B-ultrasound on 2024-12-4 showed: (1) A nodular image in the left spermatic cord (considering torsion); (2) A cystic mass in the left inguinal canal and scrotum (considering funicular hydrocele) measuring about 4.1 cm × 2.7 cm × 1.7 cm; (3) No obvious abnormalities in the right testis and epididymis (Figure 1).

**Figure 2:**
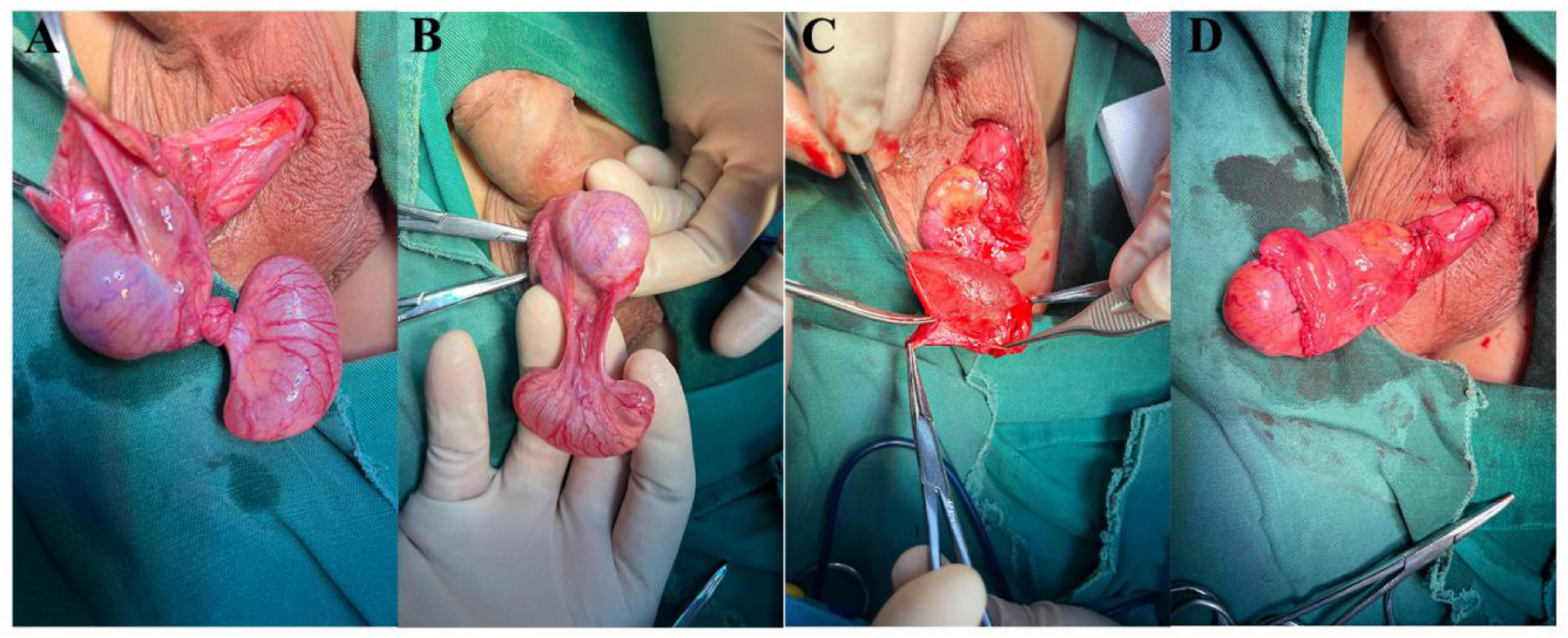
Intraoperative findings: **(A)** epididymal cyst torsion was confirmed during tunica vaginalis incision; **(B)** after reduction, a huge epididymal cyst with a long narrow pedicle was found; **(C)** complete dissection of giant cyst tissue; **(D)** the epididymis was sutured and fixed on the lateral side of the testis.

**Figure 3:**
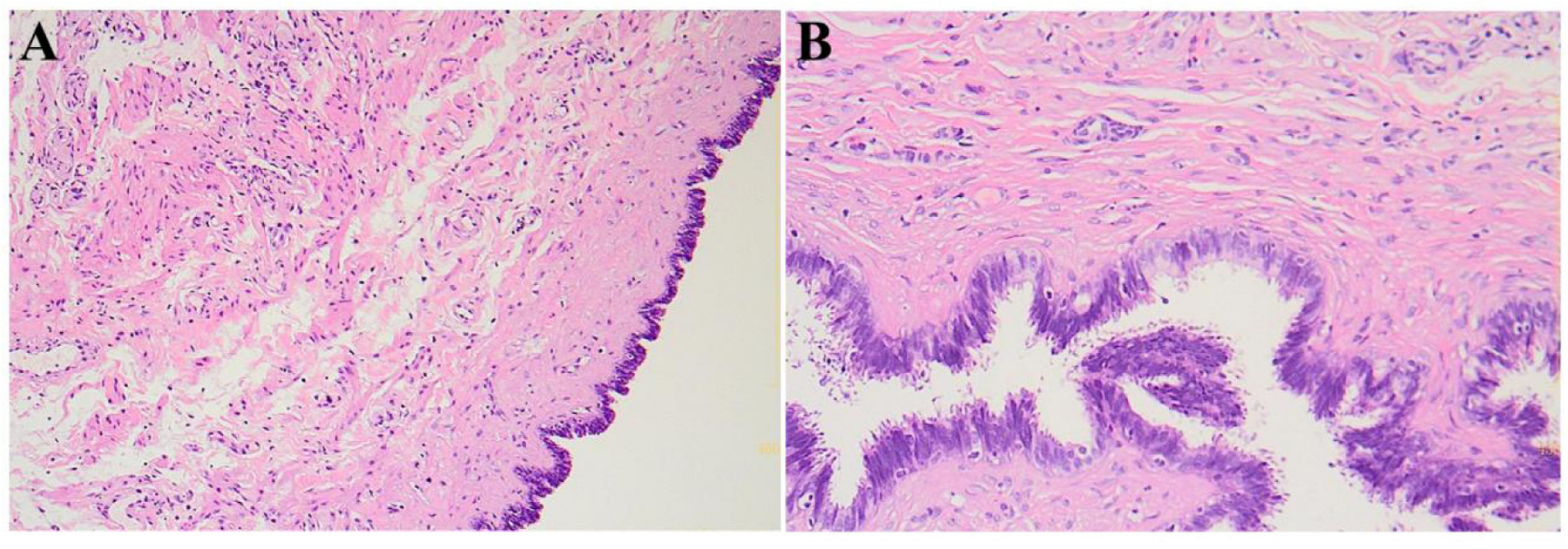
Postoperative histopathological examination; microscopically, a cystic wall-like structure is visible, with ciliated columnar epithelium lining the inner wall, and the cystic wall is composed of fibrous and smooth muscle tissue; **(A)** HE staining, 100×; **(B)** HE staining, 400×.

**Figure 4:**
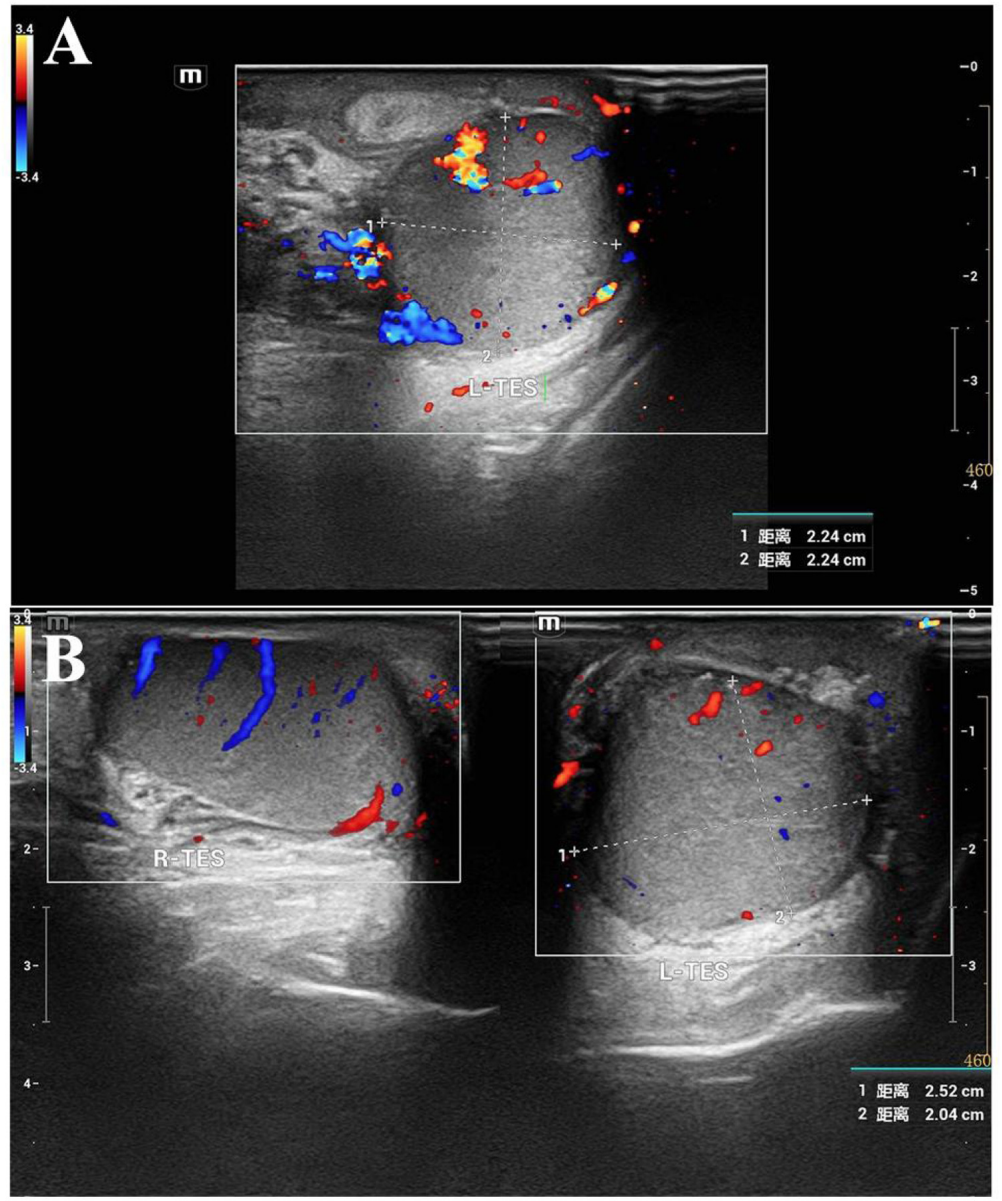
Postoperative follow-up inguinal and scrotal testicular ultrasound findings; **(A)** left testis, full shape, testis and epididymis have acceptable echo, and intra-testicular blood flow signals are visible; **(B)** comparative scanning of both testes: the left testis is slightly full, and blood flow signals are visible in both testes.

His blood tests were normal. With no history of scrotal trauma or surgery, he was considered to likely have torsion of a funicular hydrocele. After excluding anesthesia contraindications, emergency left scrotal exploration was performed. Intraoperatively, the epididymal head was cystic and enlarged, about 4.5 cm × 3.0 cm × 2.0 cm, with a twisted pedicle of about 360°. The epididymal head was red with expanded vessels but no obvious ischemia, and the testicular blood supply was normal (Figures 2A,B). After informing the family, the cyst was excised using fine instruments, and the epididymal tissue was sutured to restore normal structure and fixed to the testis. A testicular fixation was also performed. Postoperative pathology showed a cystic structure with ciliated columnar epithelium and a fibromuscular wall, indicating a left EC and possibly a mesonephric remnant cyst (Figures 3A,B). He was discharged after 2 days with good wound healing. A 3-month follow-up ultrasound showed normal blood supply to both testes and epididymides (Figures 4A,B).

## Discussion

The reported incidence of EC in children ranges from 5% to 20%, with prevalence increasing progressively with age (4). While commonly detected incidentally during routine scrotal ultrasonography as small cystic lesions, larger ECs may present as palpable cystic masses located superior to the testis on physical examination (5). The majority of pediatric EC cases remain asymptomatic, warranting conservative management. Notably, there is currently no established consensus regarding the management of incidentally discovered ECs during other testicular procedures. Spontaneous resolution occurs in up to 60% of cases, particularly when the cyst diameter is less than 3 mm (4–6).

Torsion of an EC represents an exceptionally rare clinical phenomenon that typically occurs when excessive mobility of an epididymal head cyst exists due to either laxity of the epididymo-testicular connection or anatomical variations (6). The differential diagnosis for EC torsion includes testicular torsion, epididymitis, torsion of testicular appendages, trauma, and incarcerated inguinal hernia. Additional considerations should encompass testicular tumors, idiopathic scrotal edema, and Henoch-Schönlein purpura (7, 8).

Scrotal ultrasonography serves as the diagnostic cornerstone, potentially revealing a large cystic epididymal lesion containing internal echoes and debris suggestive of hemorrhage or thrombosis. While identification of a twisted cystic pedicle provides pathognomonic evidence, this finding may not be consistently demonstrable (9). As reported by Schalamon et al., scrotal ultrasound demonstrates 84% accuracy in differentiating children with acute scrotal pain who require surgical intervention (10).

In this case, the patient presented with typical pain and scrotal erythema and swelling. Scrotal ultrasound revealed a hypoechoic cystic mass without blood flow in the left scrotum, and the patient's scrotal pain persisted. Given the possibility of testicular torsion, we decided to perform emergency surgery. Pediatric scrotal emergencies are often related to the testis, and their impact on future fertility is a major concern. Prompt and accurate diagnosis is crucial to preserve the patient's fertility. Preoperative confirmation of testicular torsion should lead to immediate surgical exploration. However, if the diagnosis is epididymitis, testicular appendage torsion, or an EC, conservative treatment with intravenous fluids may be appropriate. For EC, scrotal puncture and aspiration can be considered to avoid traumatic surgery and better protect the patient's future quality of life and fertility. This highlights the significance of reporting such cases.

The youngest reported case of epididymal cyst (EC) torsion involved a 6-month-old infant, as described by Liolios et al. (11). Our systematic search of PubMed and Web of Science using the terms “epididymal cyst” AND “torsion” AND “children” identified 12 additional cases, establishing the present case as the 13th reported instance worldwide (Table 1).

**Table 1:** Case presentation of epididymal cyst with torsion.

Case no.	Author	Year	Age (years)	Side	Initial symptoms	EC torsion degree	Treatment	Preoperative ultrasound	History of scrotal trauma	Outcome
1t	Kaye et al. (12)	1990	13	Left	Scrotal swelling and pain (chronic)	360°	Emergency surgery	EC was found	No	Rehabilitation
2nd	Liolios et al. (11)	1997	1	Left	Scrotal swelling and pain (acute)	360°	Emergency surgery	EC was not found	No	Rehabilitation
3rd	Yılmaz et al. (2)	2004	13	Left	Scrotal swelling and pain (acute)	720°	Emergency surgery	EC was found	No	Unknown
4th	Erikçi et al. (7)	2013	11	Left	Scrotal swelling and pain (chronic)	720°	Conservative treatment followed by surgery	EC was found	Yes	Rehabilitation
5th	AbdullGaffar et al. (13)	2014	14	Bilateral	Scrotal swelling	Unknown	Emergency surgery	EC was found	No	Rehabilitation
6th	Ameli et al. (14)	2015	14	Left	Scrotal swelling and pain (acute)	720°	Emergency surgery	EC was found	Yes	Rehabilitation
7th	Bleve et al. (1)	2018	16	Right	Scrotal swelling and pain (acute)	360°	Emergency surgery	EC was found	No	Rehabilitation
8th	Messina et al. (6)	2019	13	Left	Scrotal swelling and pain (acute)	Unknown	Conservative treatment followed by surgery	EC was found	Yes	Rehabilitation
9th	Ozaal et al. (15)	2020	8	Right	Scrotal swelling and pain (acute)	540°	Conservative treatment followed by surgery	EC was found	Yes	Rehabilitation
10h	Vezzali et al. (16)	2022	12	Unknown	Scrotal swelling and pain (acute)	720°	Conservative treatment followed by surgery	EC was found	No	Rehabilitation
11th	Vafadar et al. (17)	2023	11	Right	Scrotal swelling and pain (acute)	Unknown	Emergency surgery	EC was found	No	Rehabilitation
12th	Wang et al. (18)	2023	3	Right	Scrotal swelling and pain (acute)	Unknown	Conservative treatment followed by surgery	EC was found	No	Rehabilitation
This case		2025	12	Left	Scrotal swelling and pain (acute)	360°	Emergency surgery	EC was found	No	Rehabilitation

Analysis of these 13 cases revealed consistent clinical patterns. Anatomic factors predominated, with 69% (9/13) of patients demonstrating congenital epididymal-testicular laxity or an elongated cyst pedicle that predisposed to abnormal mobility (2, 6, 7). These structural variations may be aggravated by pubertal hormonal changes [69.2% of cases occurred in 10–14-year-olds (7, 13)], potentially accelerating cyst enlargement. Notably, over 80% of torsioned cysts exceeded 3 cm in diameter (1, 3, 18), suggesting that increased mass elevates pedicle tension and torsion risk. A history of trauma or vigorous activity was documented in 38.5% (5/13) of cases (4, 14, 15), supporting mechanical stress as a contributory factor.

Doppler ultrasonography emerged as the primary diagnostic tool, consistently demonstrating anechoic cystic masses in the epididymal head (13/13), with wall thickening or septations in 61.5% (8/13) (3, 5). Torsion-specific features included absent intralesional flow (76.9%, 10/13) and the whirlpool sign (53.8%, 7/13) (1, 11), while preserved testicular perfusion (13/13) distinguished EC torsion from testicular torsion (6, 18). For indeterminate cases, follow-up imaging within 4–6 h is advised to evaluate dynamic changes (10). Laboratory parameters (complete blood count, urinalysis) were universally normal (13/13), helping exclude epididymitis or urinary infections (4, 7). Key differential diagnoses include testicular torsion (absent cremasteric reflex, testicular malposition), epididymitis (Prehn's sign positivity, inflammatory markers), and testicular appendage torsion (localized pain, hyperechoic nodule) (10, 16).

Based on these characteristic findings, we recommend that clinicians first obtain a detailed history focusing on trauma, strenuous physical activity, and prior scrotal masses. This should be followed by a comprehensive physical examination assessing testicular position, cremasteric reflex, and Prehn's sign. Doppler ultrasonography should be employed as the first-line diagnostic modality, with particular attention to cyst characteristics and testicular vascularity. In cases where EC torsion is strongly suspected or when testicular torsion cannot be definitively excluded, surgical exploration within 6 h is imperative to maximize testicular salvage potential (9, 19). Notably, among all reported EC torsion cases, none of the patients who underwent emergency surgery (12/13) developed testicular ischemia or long-term complications, underscoring the critical importance of timely intervention.

Thus, EC torsion should be considered a rare cause of pediatric acute scrotal pain, especially in patients with known EC or a history of trauma. For small, asymptomatic ECs, a conservative approach is recommended. Erikci et al. suggested selective surgery for ECs larger than 10 mm. However, we do not advocate surgery for all ECs over 10 mm, especially in adolescents; a more cautious approach is needed. The decision for surgery should be based on a comprehensive assessment of the patient's specific circumstances, including the severity of symptoms, the growth rate of the cyst, its impact on quality of life, and the presence of complications. For asymptomatic or mildly symptomatic ECs, a watchful waiting strategy with regular ultrasound monitoring is appropriate. Surgery is considered when ECs cause significant symptoms such as pain or psychological stress, or when they grow rapidly and affect testicular function and development. It is also crucial to accurately recognize the signs of scrotal emergencies to avoid misdiagnosing testicular torsion as EC, which could delay necessary surgery. Additionally, when ECs are incidentally found during other testicular surgeries, it is recommended to address them surgically if it does not increase the anesthesia burden on the child.

## Conclusion

This study emphasizes EC torsion as a rare but important differential diagnosis in pediatric acute scrotal pain, distinct from testicular torsion and other scrotal conditions. Clinical examination and ultrasound are vital for diagnosis. Suspected testicular or EC torsion necessitates timely surgical exploration to prevent severe complications like testicular necrosis. The study also underscores the value of multidisciplinary collaboration in managing such complex cases, guiding future research towards more precise diagnostic criteria and treatment protocols.

## Data Availability

The original contributions presented in the study are included in the article/Supplementary Material, further inquiries can be directed to the corresponding authors.

## References

[1] Bleve C Conighi ML Bucci V Costa L Chiarenza SF. Torsion of huge epididymal cyst in a 16-year-old boy: case report and review of the literature. Pediatr Med Chir. (2018) 40(1):20–2. 10.4081/pmc.2018.162 29871476

[2] Yilmaz E Batislam E Bozdogan O Basar H Basar MM. Torsion of an epididymal cyst. Int J Urol. (2004) 11(3):182–3. 10.1111/j.1442-2042.2003.00764.x 15009369

[3] Cai W Liu C Xu L Wu Q Kuang T Lin X. Epididymal cysts in children: frequency, clinical characteristics, and management strategies. Front Pediatr. (2024) 12:1455866. 10.3389/fped.2024.1455866 39108693 PMC11300225

[4] Erikci V Hoşgör M Aksoy N Okur Ö Yildiz M Dursun A Management of epididymal cysts in childhood. J Pediatr Surg. (2013) 48(10):2153–6. 10.1016/j.jpedsurg.2013.01.058 24094972

[5] Homayoon K Suhre CD Steinhardt GF. Epididymal cysts in children: natural history. J Urol. (2004) 171(3):1274–6. 10.1097/01.ju.0000110322.87053.99 14767330

[6] Messina M Fusi G Ferrara F Bindi E Pellegrino C Molinaro F A rare cause of acute scrotum in a child: torsion of an epididymal cyst. Case report and review of the literature. Pediatr Med Chir. (2019) 41(1):22–3. 10.4081/pmc.2019.210 31232012

[7] Boscarelli A Bellini T. Epididymal cyst in children. Eur J Pediatr. (2021) 180(9):2723–9. 10.1007/s00431-021-04080-5 33851241

[8] Niedzielski J Miodek M Krakós M. Epididymal cysts in childhood—conservative or surgical approach? Pol Przegl Chir. (2012) 84(8):406–10. 10.2478/v10035-012-0068-2 22985703

[9] Ilangovan G Manimaran M Mounika V Khan MA. Torsion of epididymal cyst: a case report with review of literature. Cureus. (2023) 15(12):e51158. 10.7759/cureus.51158 38283501 PMC10811970

[10] Schalamon J Ainoedhofer H Schleef J Singer G Haxhija EQ Höllwarth ME. Management of acute scrotum in children–the impact of Doppler ultrasound. J Pediatr Surg. (2006) 41(8):1377–80. 10.1016/j.jpedsurg.2006.04.026 16863840

[11] Liolios N Anagnostopoulos D Sinopidis X Vassou N Kasselas V. Torsion of spermatocele and aplasia of the vas deferens. A case report. Eur J Pediatr Surg. (1997) 7(2):118–9. 10.1055/s-2008-1071069 9165262

[12] Kaye RI Cromie WJ. Torsion of a spermatocele: a case report and review of the literature. J Urol. (1990) 143(4):786. 10.1016/s0022-5347(17)40094-2 2313807

[13] AbdullGaffar B Al-Hasani S El-Fayomy A. Multiple bilateral nonpapillary serous cystadenoma of the epididymis. Urology. (2013) 82(3):e24–5. 10.1016/j.urology.2013.06.006 23987181

[14] Ameli M Boroumand-Noughabi S Gholami-Mahtaj L. A 14-year-old boy with torsion of the epididymal cyst. Case Rep Urol. (2015) 2015:731987. 10.1155/2015/731987 26798545 PMC4698546

[15] Ozaal AMOM Pragalathan B Lavanya S Sarma ST. Torsion of an epididymal cyst: a rare finding on scrotal exploration for acute scrotum. Case Rep Urol. (2020) 2020:8858606. 10.1155/2020/8858606 33299634 PMC7704176

[16] Vezzali N Valletta R Grandi F Neri S Ferro F. A rare case of acute scrotum in a 12-year old boy: torsion of a paradidymal cystic appendage (organ of giraldes). J Ultrasound. (2022) 25(3):591–5. 10.1007/s40477-021-00628-1 35000128 PMC9402832

[17] Vafadar M Rakhshankhah N Salek M Zarei E. Torsion of epididymal cyst as a cause of acute scrotum in a child. Urol Case Rep. (2023) 48:102417. 10.1016/j.eucr.2023.102417 37215055 PMC10192393

[18] Wang Y Wang K Yu L Ma X. A rare case of scrotal emergency: torsion of epididymal cyst-a case report and literature review. Front Pediatr. (2023) 11:1245842. 10.3389/fped.2023.1245842 38264504 PMC10803493

[19] Zhanghuang C Wang J Hang Y Ji F Yao Z Mao R A novel nomogram to predict testicular torsion in children with acute scrotal pain: a single-center retrospective study in western China. Transl Androl Urol. (2024) 13(5):776–91. 10.21037/tau-23-634 38855602 PMC11157408

